# Resistin, an Adipokine with Non-Generalized Actions on Sympathetic Nerve Activity

**DOI:** 10.3389/fphys.2015.00321

**Published:** 2015-11-10

**Authors:** Emilio Badoer, Samin Kosari, Martin J. Stebbing

**Affiliations:** School of Medical Sciences and Health Innovations Research Institute, RMIT UniversityMelbourne, VIC, Australia

**Keywords:** energy metabolism, resistin, leptin, sympathetic nerve activity, thermogenesis, brown adipose tissue, cardiovascular function

## Abstract

The World Health Organization has called obesity a global epidemic. There is a strong association between body weight gain and blood pressure. A major determinant of blood pressure is the level of activity in sympathetic nerves innervating cardiovascular organs. A characteristic of obesity, in both humans and in animal models, is an increase in sympathetic nerve activity to the skeletal muscle vasculature and to the kidneys. Obesity is now recognized as a chronic, low level inflammatory condition, and pro-inflammatory cytokines are elevated including those produced by adipose tissue. The most well-known adipokine released from fat tissue is leptin. The adipokine, resistin, is also released from adipose tissue. Resistin can act in the central nervous system to influence the sympathetic nerve activity. Here, we review the effects of resistin on sympathetic nerve activity and compare them with leptin. We build an argument that resistin and leptin may have complex interactions. Firstly, they may augment each other as both are excitatory on sympathetic nerves innervating cardiovascular organs; In contrast, they could antagonize each other's actions on brown adipose tissue, a key metabolic organ. These interactions may be important in conditions in which leptin and resistin are elevated, such as in obesity.

## Introduction

The worldwide increase in obesity is so dramatic that the World Health Organization has called obesity a global epidemic. In 2014, more than 1.9 billion adults, 18 years and older, were overweight (39%), and approximately 13% of the world's adult population were obese (WHO, 2015). Disconcertingly, the worldwide prevalence of obesity more than doubled between 1980 and 2014 (WHO, 2015). There is a close, strong association between body weight gain and blood pressure, which is similar for men and women and across age groups (Garrison et al., 1987; Hall et al., 2002; Wofford and Hall, 2004). Therefore, obesity and the metabolic syndrome increase the risk of cardiovascular disease and hypertension, and it is not surprising that with excess body weight there is increased risk of long term health consequences and a substantial increase in mortality and morbidity (WHO, 2014).

A major determinant of blood pressure is the level of activity in sympathetic nerves innervating cardiovascular organs. A characteristic of obesity, in both humans (Grassi et al., 1998; Esler et al., 2006) and in animal models (e.g., dogs, rats, and rabbits), is an increase in sympathetic nerve activity to the skeletal muscle vasculature and to the kidneys (Levin et al., 1983; Kassab et al., 1995; Prior et al., 2010). Renal sympathetic nerve activity is particularly important since it affects salt and water balance directly and indirectly by influencing renin release from the kidney (hence activation of the renin angiotensin-aldosterone system). The regulation of renal function by the sympathetic nerves is a key player in obesity induced hypertension as evidenced by the observations in obese dogs in which destruction of the nerves projecting to the kidneys prevents both sodium retention and the hypertension present in that model of obesity (Kassab et al., 1995). The causes of the elevated sympathetic nerve activity observed in obesity are unknown.

Obesity is now recognized as a chronic, low level inflammatory condition. Pro-inflammatory cytokines are elevated in obesity (Gregor and Hotamisligil, 2011) including those produced by adipose tissue, either by the fat cells or the macrophages that infiltrate the fat tissue, and are known as adipokines. The most well-known adipokine released from fat tissue is leptin. More recently, resistin, was also discovered as an adipokine released from adipose tissue.

Here we review the effects of resistin on sympathetic nerve activity and compare the effects to those of leptin, and discuss their potential interactions that may contribute to cardiovascular dysfunction.

## Resistin

Resistin is a member of the resistin-like molecule (RELM) hormone family. Two other members of the RELM family include RELM-alpha and RELM-beta. All RELM family members are characterized by 10 conserved cysteine residues. Resistin and RELM-beta contain an additional cysteine near their amino termini, which is conserved among species (Steppan et al., 2001; Steppan and Lazar, 2002). Human and rodent resistin proteins exhibit approximately 60% homology (Patel et al., 2004).

Resistin is secreted as a disulphide-linked homotrimer and circulates in plasma as either the trimer or a hexamer (Ghosh et al., 2003). The plasma levels of resistin are reported to be approximately between 7 and 14 ng/ml in humans and 36–43 ng/ml in rats (Azuma et al., 2003). In rodents, the main source of resistin is white adipose tissue and expression may vary between the different depots of adipose tissue and with gender (Steppan et al., 2001). In contrast to rodents, the expression of resistin in human adipocytes is low, and the main source of resistin in humans is macrophages (Yang et al., 2003), and in obesity, macrophages that have infiltrated into visceral white adipose tissue are the predominant source of resistin (Curat et al., 2006).

In addition to adipose tissue, resistin has also been detected in a variety of other tissues in the periphery including adrenal glands, skeletal muscle, kidney, and brown adipose tissue (BAT) (Nohira et al., 2004). The physiological role of resistin in many of these organs, however, is not clearly understood.

Resistin mRNA has been detected in the brain of rodents suggesting that endogenous production of resistin can occur in brain nuclei such as in the arcuate nucleus, ventromedial nucleus, and hippocampus (Morash et al., 2002; Wilkinson et al., 2005). In humans, resistin has been detected in cerebrospinal fluid, but the levels do not appear to correlate with changes in plasma levels, however, suggesting saturable uptake into the cerebrospinal fluid. This is possibly by an active transport mechanism across the blood brain barrier, as occurs with leptin. This, of course, does not exclude local production of resistin in the brain.

Resistin levels in plasma are elevated in obesity and diabetes. There are several studies in humans showing a positive correlation between plasma resistin levels and increased body mass index (Steppan et al., 2001; Azuma et al., 2003; Rajala et al., 2004). Although, the majority of studies confirm the correlation between resistin and obesity and Type 2 diabetes, a few reports show resistin was not increased in patients with severe insulin resistance and Type 2 diabetes (Janke et al., 2002).

### Receptors for resistin

It is surprising, perhaps, that a receptor for resistin has yet to be unequivocally identified. Recent work has suggested several candidates including a cleavage product of decorin (a proteoglycan) that may be involved in growth of white adipose tissue (Daquinag et al., 2011), adenyl cyclase associated protein-1 involved in mediating inflammatory processes in monocytes (Lee et al., 2014), and toll like receptor 4 (Tarkowski et al., 2010).

## Resistin and sympathetic nerve activity

**Figure 1:**
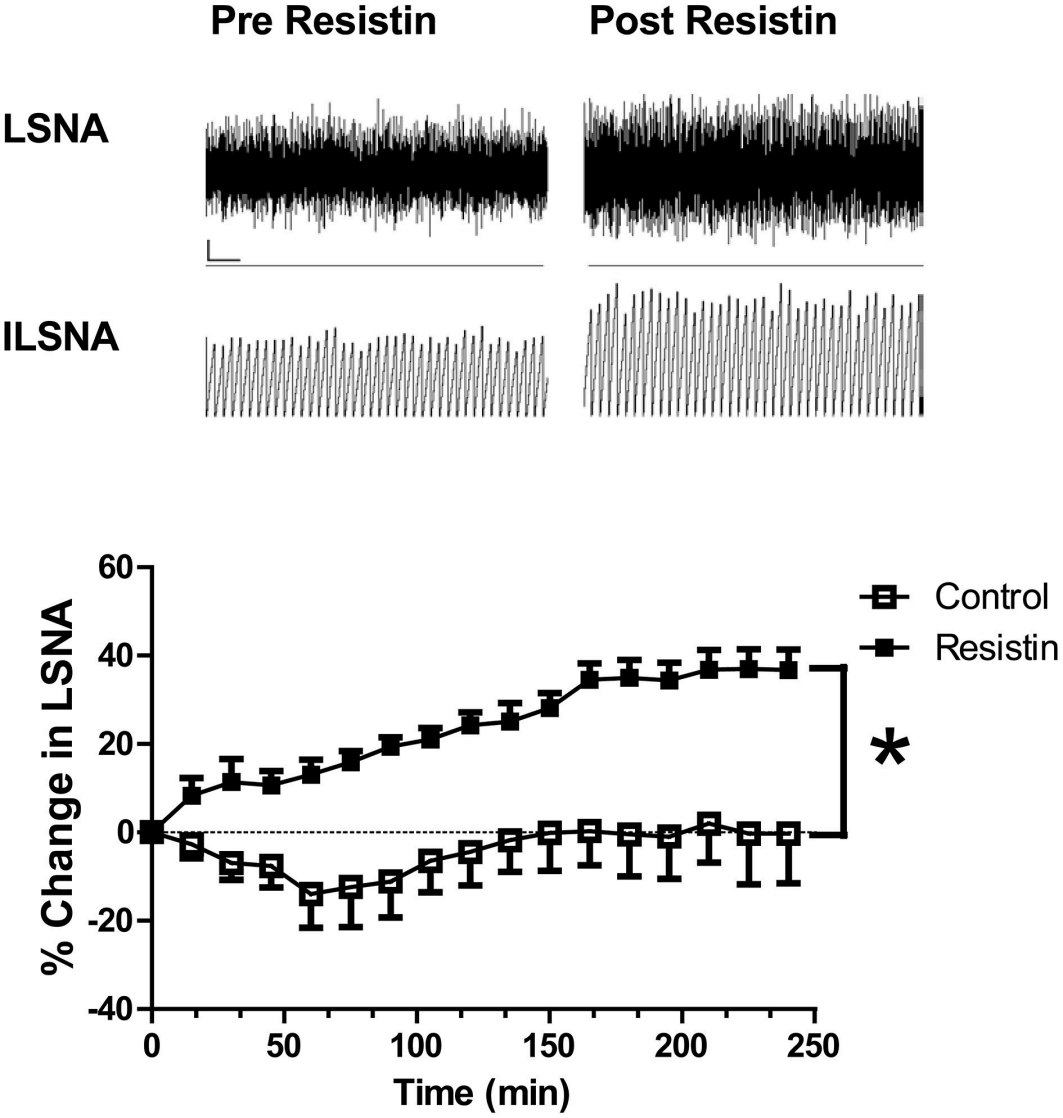
**Top:** Screen capture of the raw recordings of lumbar sympathetic nerve activity (LSNA) and integrated lumbar SNA (ILSNA) before and after resistin (7 μg) administered into the lateral brain ventricle of an anesthetized rat. ⌞, horizontal bar = 2 s, vertical bar = 100 mV (LSNA), and 10 mV.s (ILSNA). **Lower:** The percent changes in lumbar SNA from resting levels over 4 h following resistin (7 μg; *n* = 6) or control (artificial CSF; *n* = 7) administered into the lateral brain ventricle of anesthetized rats. ^*^p < 0.05 between groups Two-way ANOVA. Modified from Kosari et al. (2011).

**Figure 2:**
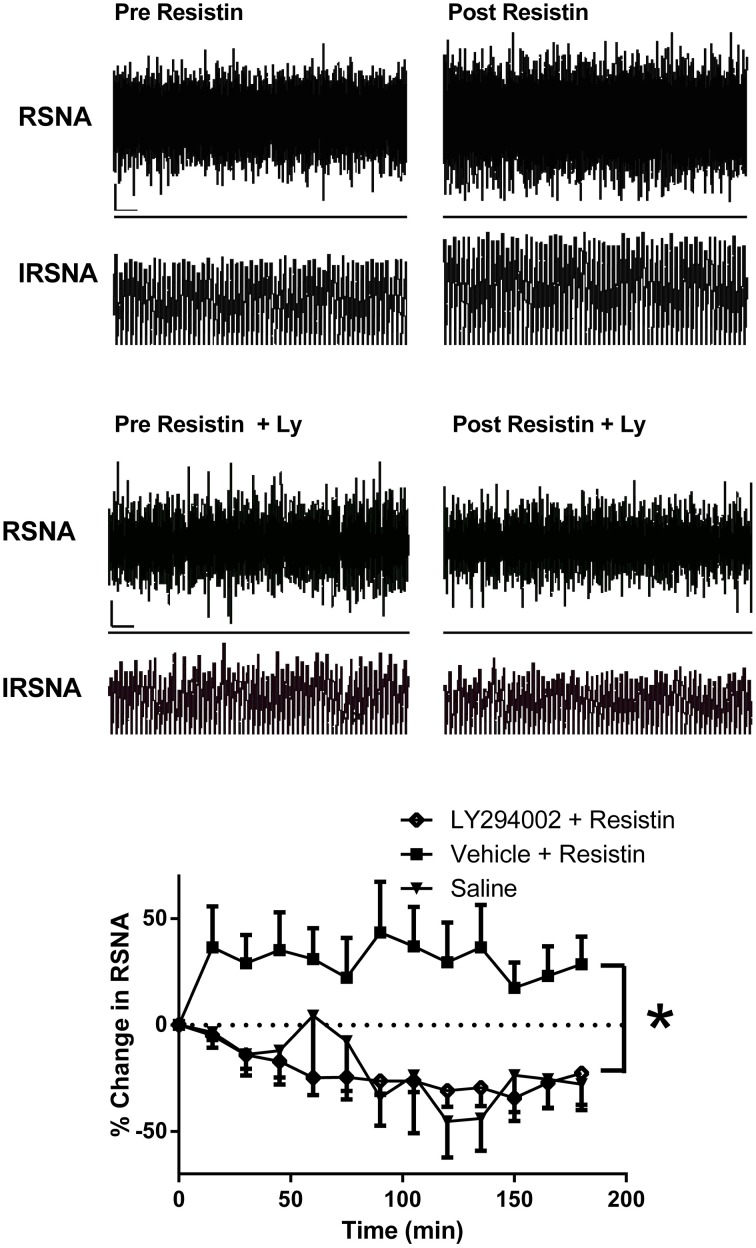
**Top:** Screen capture of raw recordings of renal sympathetic nerve activity (RSNA) and integrated renal SNA (IRSNA) before and after resistin (7 μg) administered into the lateral brain ventricle of an anesthetized rat. ⌞, horizontal bar = 2 s, vertical bar = 200 mV (RSNA), and 10 mV.s (IRSNA). Modified from Kosari et al. (2012). **Middle:** Screen capture of raw recordings of renal sympathetic nerve activity (RSNA) and integrated renal SNA (IRSNA) before and after resistin (7 μg) in the presence of the PI 3-Kinase inhibitor, LY294002 (Ly) (5 μg) administered into the lateral brain ventricle of an anesthetized rat. ⌞, horizontal bar = 2 s, vertical bar = 200 mV (RSNA), and 10 mV.s (IRSNA). Modified from Kosari et al. (2012). **Lower:** The percent changes in renal sympathetic nerve activity (RSNA) from basal levels over time following intracerebroventricular administration of saline (control) (*n* = 4) or resistin (7 μg) in the presence of LY294002, an inhibitor of PI3K (5 μg, *n* = 5) or vehicle (*n* = 7). ^*^p < 0.05 between groups, Two-way ANOVA. Modified from Kosari et al. (2012).

Acute intracerebroventricular administration of resistin induces a significant increase in lumbar sympathetic nerve activity in rats (Figure 1; Kosari et al., 2011), a measure of sympathetic nerve activity to the muscle vasculature. The effect was centrally mediated since the same dose of resistin administered intravenously failed to influence lumbar sympathetic nerve activity (Kosari et al., 2011). In a separate study investigating effects on the sympathetic outflow to the kidney in rats, resistin was also found to increase renal sympathetic nerve activity (Figure 2; Kosari et al., 2012). Since sympathetic nerve activity to the muscle vasculature and to the kidneys is elevated in obesity, metabolic syndrome, and diabetes (Grassi et al., 1998; Esler et al., 2006; Zhang et al., 2012; Coats and Cruickshank, 2014; Thorp and Schlaich, 2015), the results raised the interesting possibility that resistin may be a potential contributing factor to the cardiovascular complications associated with those metabolic conditions.

The doses of resistin utilized in reports in the literature to elicit effects on sympathetic nerve activity needs some comment. Studies have used microgram quantities administered intracerebroventricularly (Tovar et al., 2005; Singhal et al., 2007; Kosari et al., 2011, 2012, 2013) whilst, as noted above, plasma levels are in the nanogram/ml range (Azuma et al., 2003; Piestrzeniewicz et al., 2008; de Luis et al., 2009). The reasons for this difference is not immediately apparent, however, it is important to note that a similar issue is observed for leptin. With leptin microgram quantities are required intracerebroventricularly (Rahmouni et al., 2009a; Prior et al., 2010; Harlan et al., 2013; Tanida et al., 2013) to increase sympathetic nerve activity but plasma levels are in the nanogram/ml range (Aizawa-Abe et al., 2000; Bryzgalova et al., 2008; Lieb et al., 2009).

The intracellular mechanisms involved in the transduction of the renal sympathetic nerve responses elicited by resistin have been investigated. The increase in sympathetic nerve activity to the kidney elicited by resistin was prevented by the central administrations of the PI 3-kinase inhibitor LY294002 but was not affected by inhibition of ERK1/2 indicating that PI 3-kinase was an essential intracellular transduction pathway mediating the central sympatho-excitatory actions of resistin (Kosari et al., 2012; Figure 2). The central sites directly activated by resistin are not yet clearly elucidated. However, studies using the protein, Fos as a marker of increased neuronal activity, suggest the paraventricular nucleus in the hypothalamus is a potential site (Tovar et al., 2005; Singhal et al., 2007; Badoer, 2010; Kosari et al., 2011). This nucleus plays a key role in renal sympathetic nerve responses mediating body fluid regulation (Ng et al., 2004).

**Figure 3:**
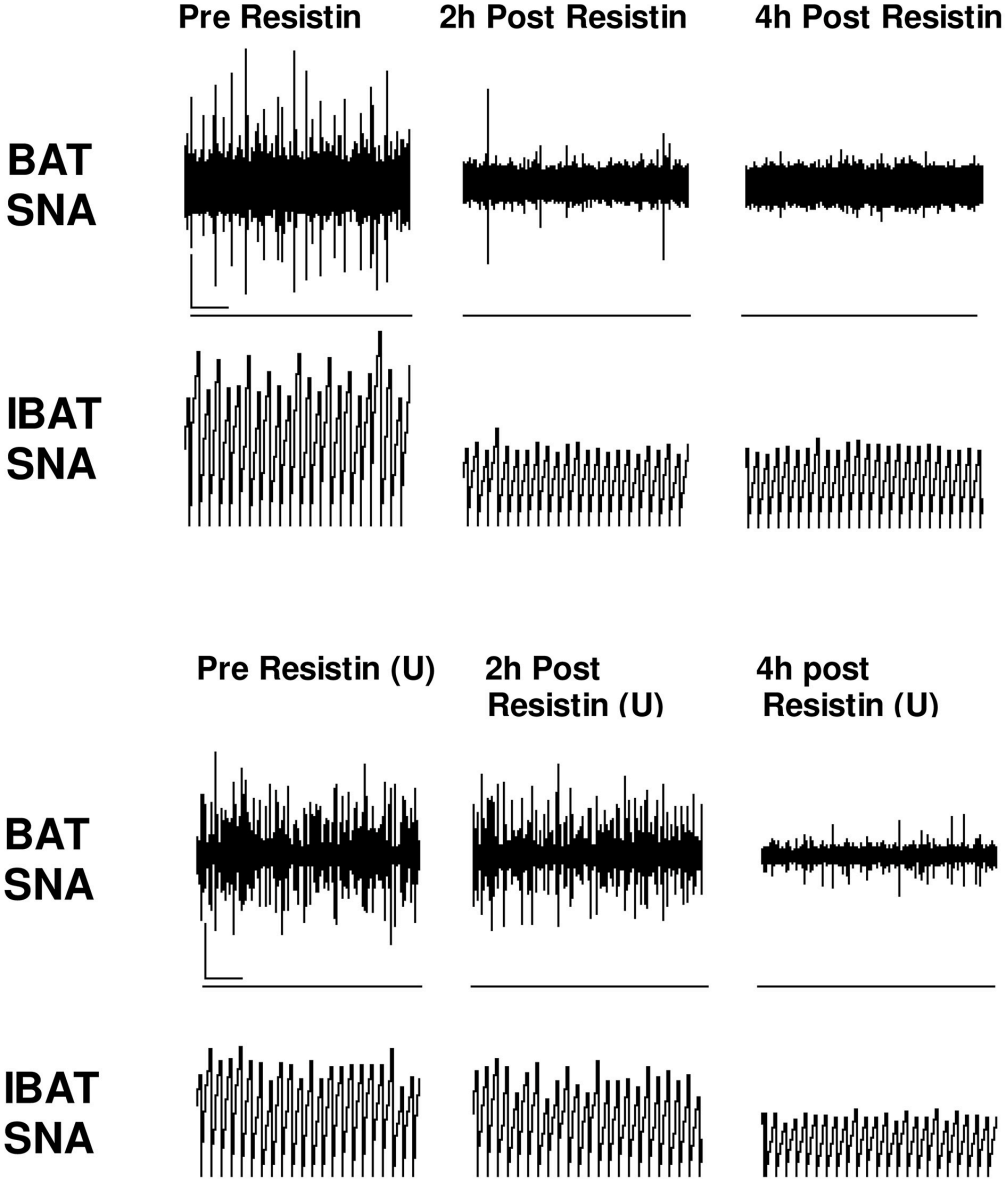
**Screen capture of raw recordings of brown adipose tissue sympathetic nerve activity (BAT SNA) and integrated BAT SNA (IBAT SNA) before, 2 and 4 h after resistin (7 μg) in the presence of vehicle or ERK1/2 inhibitor (U0126, 7 μg) (U) administered into the lateral brain ventricle of anesthetized male rats**. ⌞, horizontal bar = 2 s, vertical bar = 200 mV (BAT SNA), and 10 mV.s (IBAT SNA). Modified from Kosari et al. (2012).

Interestingly, acute intracerebroventricular administration of resistin did not significantly increase blood pressure (Kosari et al., 2011). This would be understandable if resistin's sympatho-excitatory actions to the skeletal muscle vasculature and kidneys were opposed by falls in sympathetic nerve activity to other cardiovascular organs. This has not been directly investigated to date. Alternatively, resistin may have direct sympatho-excitatory and sympatho-inhibitory actions. Supporting this argument, we have evidence that resistin does not elicit a generalized increase in sympathetic nerve activity. In contrast to the excitatory actions of resistin on lumbar and renal sympathetic nerve activities, resistin, administered into the lateral cerebral ventricle of the anesthetized rat induced, a reduction in sympathetic nerve activity to brown adipose tissue (Kosari et al., 2011; Figure 3). Brown adipose tissue is an important thermoregulatory organ. In humans it was originally thought to be lost with age but it is now known to be present and functionally active in adult humans (Nedergaard et al., 2007). The data suggests resistin has inhibitory actions on thermogenesis. Thus, resistin has differential effects on sympathetic outflow; excitatory to some organs and inhibitory on others.

To date, studies investigating the effects of resistin on blood pressure have studied the effects over short time frames (up to 4 h; Kosari et al., 2011, 2012). Whether, chronic administration of resistin has effects on blood pressure and heart rate also needs to be investigated, particularly since leptin has similar sympatho-excitatory effects to resistin on lumbar and renal sympathetic nerve activity (see later), and effects on blood pressure also differ when acute and chronic administration of leptin are compared (Beltowski, 2006). Since renal function is critical to chronic blood pressure regulation, if the acute effects of resistin on renal sympathetic nerve activity are maintained chronically, there may indeed be long term effects that could lead to elevated blood pressure in metabolic conditions in which resistin levels are elevated.

The intracellular mechanisms that contribute to the central effect of resistin on sympathetic nerve activity to brown adipose tissue involves ERK 1/2 dependent pathways since the effects are attenuated by the ERK 1/2 inhibitor U0126. Inhibition of PI 3-kinase, however, had no effect on the sympatho-inhibitory action of resistin (Kosari et al., 2012). Thus, the intracellular transduction pathways in the brain mediating the sympatho-excitatory and sympatho-inhibitory responses are different.

### Hypertension and resistin

Since resistin was found to be sympatho-excitatory to key cardiovascular tissues, one may have expected it to induce an increase in blood pressure. As indicated earlier, this has not been observed following acute administration of resistin in rats. One possibility may be that resistin needs to be administered chronically to observe increases in blood pressure. This has not been investigated, however, several clinical studies have shown that plasma resistin levels are associated with either the presence or the development of high blood pressure in humans. A positive correlation between plasma resistin levels and hypertension, measured using 24 h ambulatory blood pressure monitoring, has been observed (Papadopoulos et al., 2009; Thomopoulos et al., 2010). Increased plasma resistin levels has also been observed in a young healthy population with a family history for essential hypertension (Papadopoulos et al., 2008). This finding suggests that plasma resistin levels may have a predictive value. This view has been supported by the findings that plasma resistin levels were correlated with the risk of developing hypertension over a 14 year follow up of 872 women without previous history of hypertension or diabetes (Zhang et al., 2010). Thus, the vast majority of studies have shown a correlation between plasma resistin levels and hypertension (Ellington et al., 2007; Takata et al., 2008; Dimitriadis et al., 2009; Papadopoulos et al., 2009; Thomopoulos et al., 2010; Zhang et al., 2010). By contrast only a few studies did not observe a significant correlation (Furuhashi et al., 2003) or the correlation was observed in patients with other risk factors present, such as Type 2 diabetes, making the interpretation difficult (Asgary et al., 2014).

## Leptin

Leptin, discovered by Friedman and colleagues in 1994 (Zhang et al., 1994), is the most investigated adipokine produced by the adipose tissue. Leptin levels are increased in obesity in proportion to fat mass and leptin is well-known to act in the central nervous system to reduce food intake by regulating neuropeptides in the hypothalamus; for example by reducing the activity of neurons containing neuropeptide-Y (NPY) and agouti-related protein (AgRP) and stimulating pro-opiomelanocortin (POMC)/cocaine and amphetamine regulated transcript (CART) neurons in the arcuate nucleus of the hypothalamus (Elias et al., 1998). Considerable evidence indicates that leptin has also sympatho-excitatory actions.

### Leptin and sympathetic nerve activity

Leptin administered acutely in the lateral cerebral ventricles increases sympathetic nerve activity to the kidney, skeletal muscle vasculature, splanchnic region, adrenal gland, and brown adipose tissue (Dunbar et al., 1997; Haynes et al., 1997). Thus, leptin appears to have a generalized action in increasing sympathetic nerve activity. The sympatho-excitatory actions of leptin are primarily mediated via the activation of receptors in the brain, although recent evidence suggests that leptin can also activate afferent nerve activity from white adipose tissue that results in increased sympathetic nerve activity (Xiong et al., 2012).

The mechanism of action and chemical mediators in the brain that contribute to the increase in sympathetic nerve activity induced by leptin have been investigated. Blockade of melanocortin 3/4 receptors, histamine H1 receptors and angiotensin 1A receptors have been found to attenuate or prevent the actions of leptin (da Silva et al., 2004; Morrison, 2004; Hilzendeger et al., 2012). These data suggest that leptin, in addition to the activation of its own receptors, also activates central pathways that utilize melanocyte stimulating hormone, histamine, and angiotensin II and these participate in the ability of leptin to increase sympathetic nerve activity.

The intracellular transduction pathways involved in the sympatho-excitatory responses induced by leptin involve PI 3-Kinase, ERK1/2, and MTORC1. However, the contributions of each of these may depend on the central pathways utilized to influence the specific end organs. The increase in sympathetic nerve activity to the kidney involves activation of PI 3-kinase (Rahmouni et al., 2003, 2009a), but this is not the case for lumbar and adrenal sympathetic nerve activities. Furthermore, sympathetic nerve activity subserving brown adipose tissue involves ERK 1/2 (Rahmouni et al., 2009b). Thus, although leptin appears to induce a generalized increase in sympathetic nerve activity, there is a clear demonstration of different central pathways utilized by leptin to elicit increases in sympathetic nerve activity to different end organs. Whether, this involves different neurons within the same brain nuclei and/or different nuclei subserving different responses requires investigation.

The arcuate nucleus, containing POMC neurons, appears to be important in both the metabolic, and sympathetic responses to leptin. Increases in sympathetic nerve activity to the kidneys and to brown adipose tissue can be elicited by leptin acting in the arcuate nucleus (Rahmouni and Morgan, 2007; Harlan et al., 2011). There is some evidence, however, that different nuclei contribute to increases in sympathetic nerve activity to different end organs. For example, the subfornical organ (SFO) and nucleus tractus solitarius (NTS); the SFO is a forebrain structure lining the anterior wall of the third ventricle and lacks a blood brain barrier, making it accessible to systemic leptin. Selective removal of leptin receptors from the SFO by targeted microinjection into the SFO of adenovirus encoding Cre-recombinase in ObR(flox/flox) mice prevented the increase in renal sympathetic nerve activity but was without effect on leptin-induced increases in sympathetic nerve activity supplying brown adipose tissue (Young et al., 2013). Local injections of leptin into the NTS increased renal but was without effect on brown adipose tissue sympathetic nerve activities (Mark et al., 2009). It should be noted, however, that indirect measures of sympathetic nerve activity to brown adipose tissue, such as core body temperature, has been reported to increase following leptin administration into the fourth ventricle that overlies the NTS (Skibicka and Grill, 2009). Receptors for leptin are also found in other autonomic nuclei in the brain and activation of these by leptin can induce increases in sympathetic nerve activity. These nuclei include the rostral ventrolateral medulla, dorsomedial, and ventromedial hypothalamus (Montanaro et al., 2005). Interestingly, the dorsomedial hypothalamus appears to be essential in mediating leptin's response in sympathetic nerve activity to brown adipose tissue without affecting renal sympathetic nerve activity (Zhang et al., 2011). Nonetheless, leptin receptors in the dorsomedial hypothalamus appear to be important contributors to obesity-associated hypertension (Simonds et al., 2014). Thus, leptin may act on several brain nuclei to elicit its sympathetic nerve activity responses. Exactly, how these inter-relate or are accessed and/or activated by systemic leptin awaits further investigation. It appears, however, that some nuclei are responsible for specific sympathetic outputs whilst others may integrate several sympathetic outputs.

#### Leptin sympathetic nerve activity and obesity

The elevation in sympathetic nerve activity induced by leptin is believed to contribute to obesity induced hypertension. Studies in rodents and rabbits in which administration of leptin chronically increased renal sympathetic nerve activity and blood pressure and studies showing that the increased blood pressure induced by a high fat diet was maintained by leptin strongly support this view (Beltowski, 2006; Lim et al., 2013; Simonds and Cowley, 2013; Prior et al., 2014). The role of leptin is further strengthened by studies showing that in rodents that lack leptin or its receptor do not become hypertensive when fed high fat diets (Simonds et al., 2014). However, evidence in humans is not so forthcoming, primarily due to the lack of studies specifically designed to investigate this issue. In animals, although, acute injection of leptin does not consistently raise blood pressure, despite increasing sympathetic nerve activity, chronic infusion of leptin does increase blood pressure via sympathetically mediated changes (Beltowski, 2006). Such a response could account for the sustained increases in blood pressure that can accompany obesity. This was suggested by the early studies comparing obese leptin deficient rodents which had lower blood pressure than their lean controls (Mark et al., 1999), and by studies in which infusion of leptin into obese animals lacking leptin raised blood pressure (Aizawa-Abe et al., 2000). Thus, leptin appeared to be an important contributor to hypertension that may accompany obesity.

Genetically engineered obesity models, however, are quite different from models of obesity induced by diet. Here too, however, the evidence for a role of leptin in hypertension is relatively strong in animal studies (Prior et al., 2010; Simonds et al., 2014). Recent work in rabbits is particularly interesting since it suggests that not only is leptin responsible for the increased sympathetic nerve activity and hypertension observed with high fat feeding, but that the effect can last even when the high fat diet is removed and replaced with a normal diet which restored plasma leptin levels back to normal (Armitage et al., 2012). This suggests that leptin may initiate hypertension and may contribute to changes that can maintain high blood pressure.

#### Obesity and leptin sensitivity

It has been known for some time that the ability of leptin to reduce food intake was sharply attenuated in obese conditions. This attenuation of the anorectic actions of leptin are due to decreased receptor sensitivity at sites that are critical to food intake (Mitchell et al., 2009). Thus, in obesity the effects of leptin on food intake are weaker than in the lean state and this provided an explanation as to why in obesity, food intake was not reduced as expected given the markedly elevated plasma levels of leptin.

Interestingly, estimates from clinical studies have suggested about half the correlation between blood pressure and body weight can be attributed to the variance in leptin levels in humans (Abramson et al., 2011). How could leptin be responsible for a relatively large proportion of the obesity-induced hypertension? Such a question was perplexing when the metabolic/dietary effects of leptin were clearly attenuated in obese conditions. One explanation is the concept of selective leptin resistance introduced by Mark et al. (2002). Originally, this concept was derived from observations in models of obesity, in which the authors determined that the blood pressure responses elicited by leptin were similar in obese and lean conditions which contrasted with the different dietary intake responses elicited by leptin in those conditions.

The original findings used renal sympathetic nerve activity as the neural output to show that the sensitivity to leptin was retained in obesity. Subsequent findings, however, showed that the response in sympathetic nerve activity innervating brown adipose tissue following leptin was attenuated in obese compared to lean animals (Rahmouni et al., 2005). Such results suggested that there was differential resistance to leptin's actions on sympathetic nerve activity in obesity. The resistance to the effects of leptin on sympathetic nerve activity to brown adipose tissue has been questioned recently in experiments in which the increase in temperature of brown adipose tissue in response to leptin (a indirect marker of sympathetic nerve activity to that tissue) was not attenuated in diet-induced obese mice compared to lean controls (Enriori et al., 2011).

There is also evidence of differential selective resistance between the renal sympathetic nerve activity and sympathetic outflows to other cardiovascular organs. For example, in contrast to the renal sympathetic nerve activity, the responses to leptin in both lumbar and splanchnic sympathetic nerve activities are attenuated in obese conditions compared to lean controls (Rahmouni et al., 2005). However, since the kidneys are critical organs involved in cardiovascular function and chronic blood pressure maintenance, the resistance to attenuation of the renal sympathetic nerve response elicited by leptin, in the obese condition could make a critical contribution to obesity-induced hypertension.

### Comparison of the effects of leptin and resistin on sympathetic nerve activity

Based on the data to date, leptin and resistin increase sympathetic outflow to the renal and lumbar regions (Table 1). The intracellular transduction pathways mediating the changes in renal sympathetic nerve activity are similar in that both utilize PI 3-Kinase. Whether, the same neurons are utilized awaits investigation. The effect of resistin on brown adipose tissue sympathetic nerve activity, however, is opposite to that induced by leptin, indicating the two have opposing actions on thermogenesis.

**Table 1:** **Comparison of effects of resistin and leptin on blood pressure and sympathetic nerve activity (SNA) to different cardiovascular and thermogenic outputs**.

**Output**	**Resistin**	**Species and references**	**Leptin**	**Species and references**
Blood pressure	No change	Rat (Kosari et al., 2011, 2012)	No change	Rat (Beltowski, 2006)
			↑	Rat (Dunbar et al., 1997; Beltowski, 2006)
Renal SNA	↑	Rat (Kosari et al., 2012)	↑	Rat (Dunbar et al., 1997; Haynes et al., 1997; Rahmouni et al., 2009a)
				Rabbit (Prior et al., 2010)
				Mice (Rahmouni et al., 2003)
Lumbar SNA	↑	Rat (Kosari et al., 2011)	↑	Rat (Dunbar et al., 1997)
Splanchnic SNA	Unknown		↑	Rat (Haynes et al., 1997)
BAT SNA	↓	Rat (Kosari et al., 2013)	↑	Rat (Rahmouni et al., 2009b)

*Arrow direction indicates increase or decrease*.

Given our discussion above on the importance of the renal sympathetic nerve responses in the potential for leptin to contribute to obesity-induced hypertension, the similar sympatho-excitatory actions of resistin and leptin on renal sympathetic nerve activity could assume greater significance. We are currently exploring the possibility that leptin and resistin could have enhanced actions on renal sympathetic nerve activity when both adipokines are present. Additionally, it is not known whether the selective resistance to the responses of leptin on renal sympathetic nerve activity observed in the obese condition, also occurs with resistin. If this is the case, then this could be significant with respect to obesity–induced hypertension, since plasma levels of both adipokines are elevated in obesity.

#### Perspective

**Figure 4:**
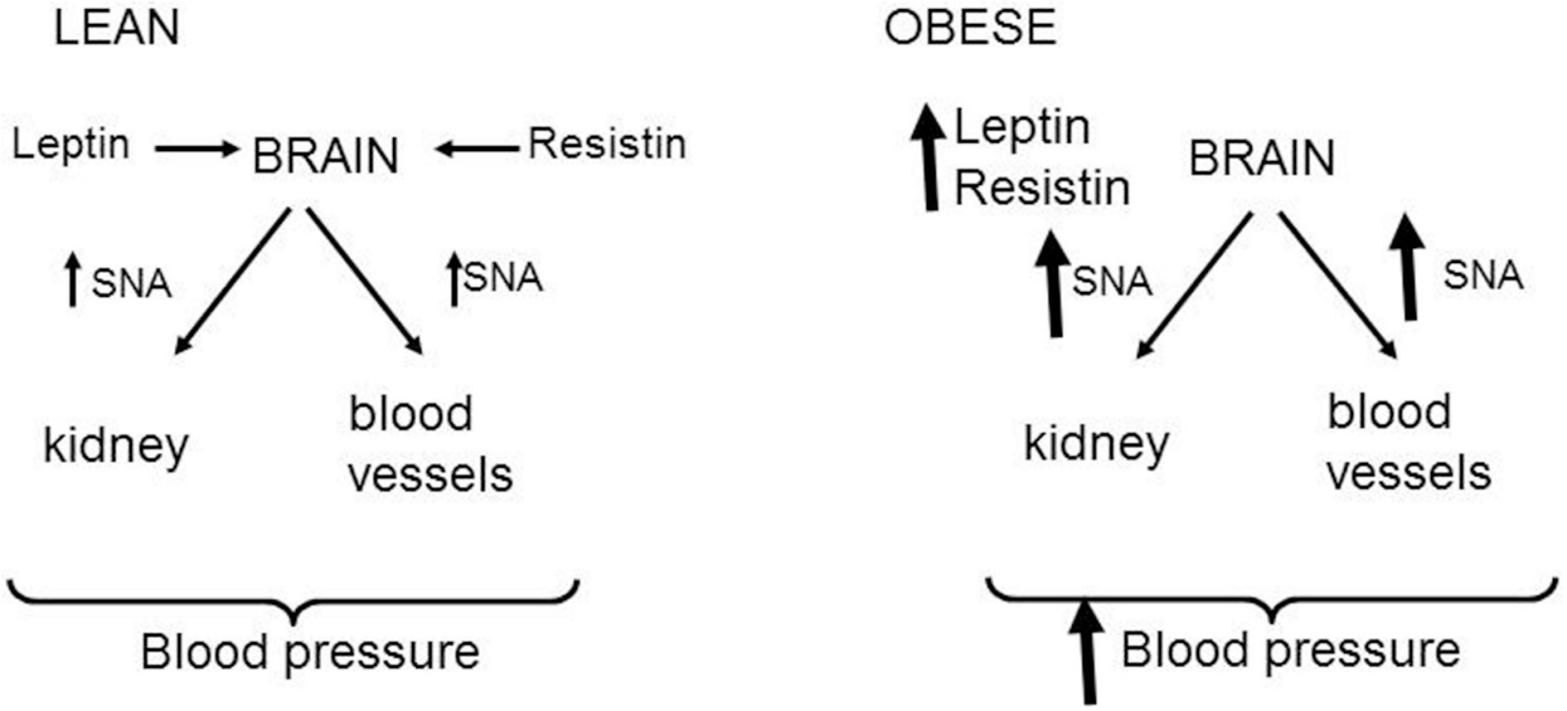
**Schematic representation of the effects of leptin and resistin on sympathetic nerve activity (SNA) to cardiovascular organs**. In normal lean states, resistin, and leptin act in the brain to increase sympathetic nerve activity to the kidney and skeletal muscle vasculature. These may influence blood pressure. In the obese state, it is hypothesized that the increased levels of leptin and resistin leads to enhanced levels of sympathetic nerve firing to the kidney and skeletal muscle vasculature that could contribute to obesity-induced hypertension.

In the obese condition, there is an elevation in renal and skeletal muscle sympathetic nerve activity, and the increase in muscle sympathetic nerve activity has been found to correlate with the amount of visceral fat tissue (Alvarez et al., 2002). The findings that resistin and leptin increase renal and lumbar sympathetic nerve activity, taken together with the reports that plasma levels of leptin and resistin are elevated in obesity, suggests that resistin and leptin could contribute to the sympathetic nerve activity disturbances observed in obesity (Figure 4). Evidence that leptin contributes to the elevated sympathetic nerve activity in obese conditions supports such a contention but resistin's role needs to be investigated. Nonetheless, as both resistin and leptin are elevated in obesity, and they each have sympatho-excitatory actions on renal and lumbar sympathetic nerve activity, it is quite possible that the effects on those outputs is exacerbated when leptin and resistin are present together. An exacerbated effect on renal sympathetic nerve activity could influence sodium retention, renal haemodynamics, and long term regulation of blood pressure, all of which are abnormally elevated in obesity. Thus, we hypothesize that leptin and resistin, when present in high concentrations, may act together and contribute to obesity-induced hypertension and renal dysfunction. Further, studies in animal models of obesity are needed to explore this hypothesis.

**Figure 5:**
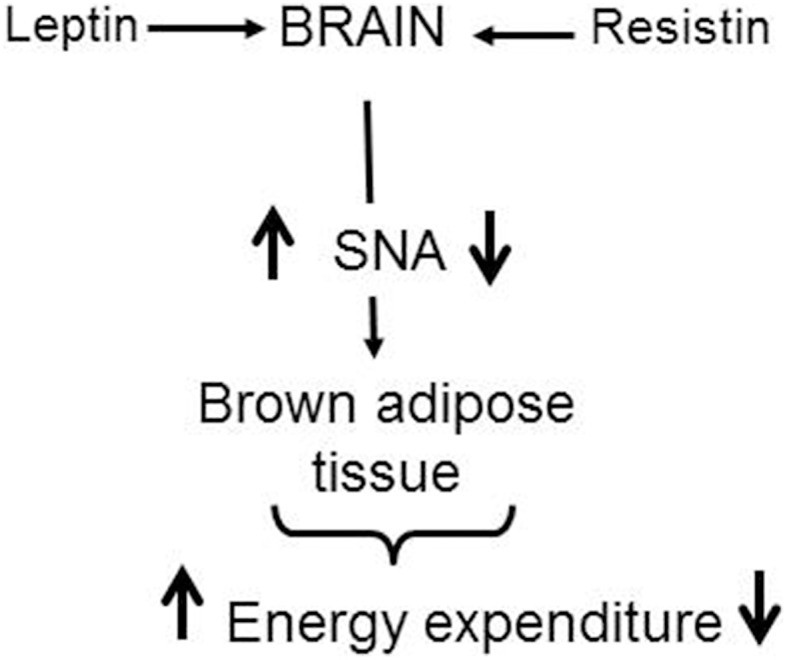
**Schematic representation of the central effects of leptin and resistin on sympathetic nerve activity (SNA) to brown adipose tissue**. This contributes to changes in energy expenditure. Leptin increases SNA to brown adipose tissue resulting in increased thermogenesis and energy expenditure. In contrast, resistin reduces SNA to brown adipose tissue resulting in a reduction in thermogenesis. In obesity when leptin and resistin levels are elevated, it is tempting to speculate that the interaction with resistin could contribute to the reduced leptin effects observed on SNA to brown adipose tissue.

In contrast to the similar sympatho-excitatory effects of resistin and leptin on renal and lumbar sympathetic nerve activity, resistin reduces, whilst leptin increases, sympathetic nerve activity innervating brown adipose tissue. The contrasting actions on thermogenesis suggests resistin could blunt leptin's action on this metabolic output (Figure 5). Support for this idea has recently been found in a study from Scherer's group (Asterholm et al., 2014). Based on the findings, it is tempting to speculate that an interaction with resistin could contribute to the selective leptin resistance observed on sympathetic nerve activity to the kidney but not to brown adipose tissue.

## Author contributions

EB conceived, designed, supervised, and interpreted the work and wrote the manuscript. SK performed the original experiments, collected, and interpreted the data and contributed to the manuscript. MS contributed to interpretation of the data and contributed to the manuscript.

### Conflict of interest statement

The authors declare that the research was conducted in the absence of any commercial or financial relationships that could be construed as a potential conflict of interest.
